# Computer Vision-Based Construction Process Sensing for Cyber–Physical Systems: A Review

**DOI:** 10.3390/s21165468

**Published:** 2021-08-13

**Authors:** Binghan Zhang, Bin Yang, Congjun Wang, Zhichen Wang, Boda Liu, Tengwei Fang

**Affiliations:** 1 Department of Structural Engineering, Tongji University, 1239 Siping, Shanghai 200092, China; zhangbinghan@tongji.edu.cn (B.Z.); wangzhichen@tongji.edu.cn (Z.W.); W-lbd-W@tongji.edu.cn (B.L.); Kevinfang@tongji.edu.cn (T.F.); 2 Zhongyifeng Construction Group Co., Suzhou 215131, China; wangcongjun@zyfchina.com

**Keywords:** computer vision, cyber–physical systems, sensing system, review

## Abstract

Cyber–physical systems (CPSs) are generally considered to be the next generation of engineered systems. However, the actual application of CPSs in the Architecture, Engineering and Construction (AEC) industry is still at a low level. The sensing method in the construction process plays a very important role in the establishment of CPSs. Therefore, the purpose of this paper is to discuss the application potential of computer vision-based sensing methods and provide practical suggestions through a literature review. This paper provides a review of the current application of CPSs in the AEC industry, summarizes the current knowledge gaps, and discusses the problems with the current construction site sensing approach. Considering the unique advantages of the computer vision (CV) method at the construction site, the application of CV for different construction entities was reviewed and summarized to achieve a CV-based construction site sensing approach for construction process CPSs. The potential of CPS can be further stimulated by providing rich information from on-site sensing using CV methods. According to the review, this approach has unique advantages in the specific environment of the construction site. Based on the current knowledge gap identified in the literature review, this paper proposes a novel concept of visual-based construction site sensing method for CPS application, and an architecture for CV-based CPS is proposed as an implementation of this concept. The main contribution of this paper is to propose a CPS architecture using computer vision as the main information acquisition method based on the literature review. This architecture innovatively introduces computer vision as a sensing method of construction sites, and realizes low-cost and non-invasive information acquisition in complex construction scenarios. This method can be used as an important supplement to on-site sensing to further promote the automation and intelligence of the construction process.

## 1. Introduction

With the continuous development of computer science, researchers have become increasingly interested in the use of digital virtual technologies in the construction industry. Due to the growing demand for information exchange on the construction site, cyber–physical system (CPS) architecture has become a highly promising approach. Cyber–physical systems (CPSs) refer to the integration of physical processes and computation [1,2,3]. CPSs can be considered as a confluence of distributed sensor systems and controls [4]. It is a transformative technology for managing interconnected systems between its physical assets and computational capabilities [5]. In order to achieve bi-directional coordination, computational resources are needed to tightly integrate the virtual and physical domains [5]. Using a CPS can bring great social and economic benefits. Many developed industrial countries, represented by the United States and European Union, have already turned their attention to the research of CPSs and provided enormous investment [6].

However, the application of the current CPS architecture on the construction site still faces many limitations. Although the widespread application of building information modeling (BIM) technology in construction projects has greatly facilitated the establishment of the cyber side of CPSs, how to achieve data exchange and coordination from the physical side to the cyber side based on multiple sensors is still a major problem due to the decentralized and complex nature of construction sites. Cost is also a problem in the application of CPS in construction sites. Contractors still have doubts about the benefits of their investment at the CPS level. On the other hand, at the researcher, professional, and institutional levels, barriers have resulted in narrowly defined, discipline-specific research and education venues in academia for the science and engineering disciplines. Research of CPSs is partitioned into isolated sub-disciplines [7]. Therefore, a novel approach of CPS needs to be proposed. This approach should be applied in changing and complex scenarios and sense the construction process information. Based on these points, computer vision is taken into consideration as a complement to CPS’s ability to sense the physical world.

Computer vision (CV) allows computers to obtain high-level, abstract, and computable information from images or video. Currently, deep learning-based CV methods are widely accepted. Since the demonstration of convolutional neural networks (CNN) for image classification tasks in 1998 [8], this approach has been continuously developed. In 2006, the concept of deep learning was significantly developed [9]. Then, at the 2012 ImageNet competition, deep convolutional networks have almost halved the error rates of the best competing approaches [10,11]. At the same time, the improvement in computing performance has further stimulated the development of computer vision methods based on CNN. A variety of deep learning and CNN-based algorithms have been developed for major tasks in computer vision including image classification, object detection, object tracking, instance segmentation, semantic segmentation, etc. From an engineering perspective, computer vision can automate tasks that require human vision. Studies have been conducted on vision-based video or photographic analysis and image processing techniques for progress monitoring and scheduling, tracking equipment operation, and distinguish construction equipment [12].

The innovation and development of CPS will require computer scientists and network professionals to work with experts in various engineering disciplines including control engineering, signal processing, civil engineering, etc. [4]. These advances at the technical level have made CV-based CPS possible and provide a new approach to cyber–physical bi-directional information exchange. Compared to traditional CPS sensing methods (e.g., sensor networks [13], IoT [14], RFID-RTLS), CV-based technology for sensing offers completely new possibilities for CPS. This approach has advantages, on one hand, at the cost level, and on the other hand, there are better solutions for the decentralized nature of construction sites. In addition, the computer vision method aims to automate the work that can be done by human vision. For the traditional manual construction site management method, the CV method can provide a foundation for the automation and intelligence in construction sites.

In this paper, a review of the state-of-the-art applications of CPS in the construction industry is presented, along with an analysis of the currently existing knowledge gaps that affect the further application of CPS on the construction site. Afterward, based on the review of computer vision technology application in construction, a method to establish a bridge for the transmission of information from the physical side to the cyber side of CPS using the computer vision method is proposed. For the application of CPS in the construction industry, this paper focuses more on the combination of the construction process and CPS to enable better intelligent management of this important phase of construction. This CPS framework focuses on the construction process, and innovatively introduces computer vision as a sensing method of the construction site, and realizes low-cost and non-invasive information acquisition in complex construction scenarios. The feasibility of this method was further analyzed. Finally, the implementation method of a computer vision-based CPS system is discussed and conclusions are drawn. This method can be used as an important supplement to on-site sensing to further promote the automation and intelligence of the construction process.

## 2. Methods

This article conducts an overview study of CPS applications at current construction sites. Since the sensing of the physical environment is an important part of CPS, and one of the main obstacles to the current application of CPS on construction sites is that site sensing is affected by complex environments, this paper is dedicated to a CV-based approach to site sensing. For these reasons, the scope of the literature in this paper covers two main areas: the CPS and the application of CV in construction site sensing.

This paper collects and analyzes the literature from academic publications on the application of CPS in the construction industry and the application of CV methods to perceive construction behavior on the construction site. The methodology used in this paper refers to the collection and analysis of the literature in [15,16]. The methodology of this paper consists of the following four steps: (1) Select the keywords for literature searching based on the research scope; (2) literature collection and organization; (3) analyze the contents of the literature and summarize the state of application of CPS and CV in construction sites under current technical conditions, and identify the knowledge gaps between current methods to an applicable CPS for construction sites; and (4) according to the existing problems and needs of the current construction site, combined with the knowledge gap in the research field, suggestions and solutions are put forward to better improve the intelligent level of the construction site of the construction industry in the era of Industry 4.0.

Of these, steps 1 and 2 will be discussed in this section, and the collected literature will be initially organized and analyzed. Step 3 will be analyzed and summarized in detail in Section 3 and Section 4. Step 4 will be further developed in Section 5.

**Figure 1:**
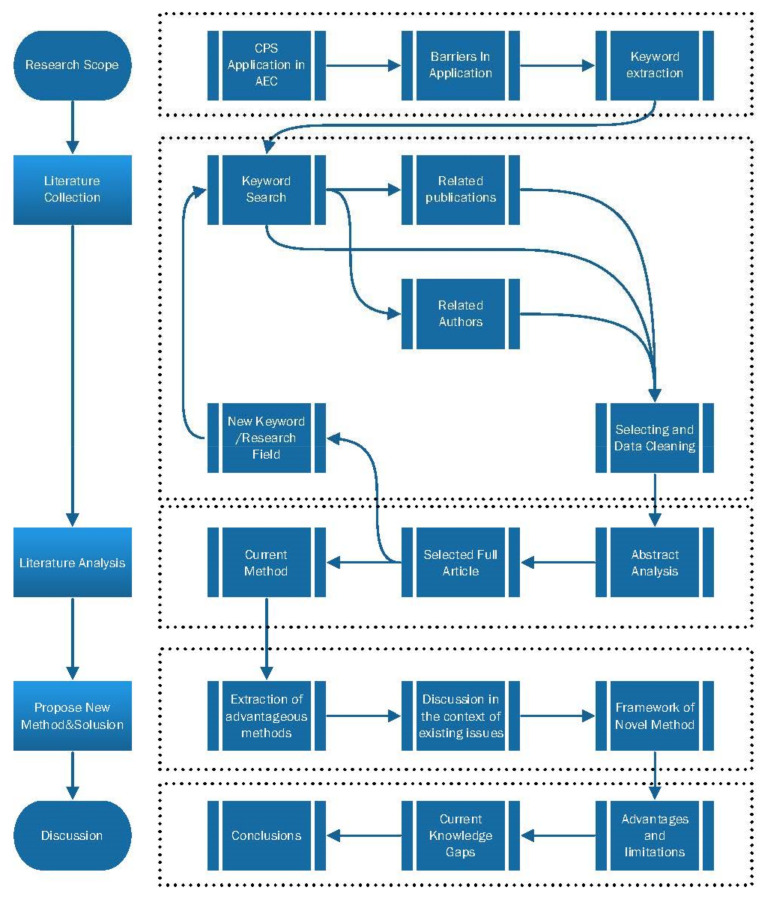
Research roadmap.

The procedure of this research can be seen in Figure 1. Based on this process, the topic of this paper was identified. At the same time, the cyclic of keywords—literature collection—organization—new keywords is used for the problems and possible solutions of CPS in construction site applications.

The literature collected included two main parts: cyber–physical system(s) and computer vision. Both were narrowed down in research areas to “CONSTRUCTION BUILDING TECHNOLOGY.” After keyword searching, the literature was further filtered based on the title and abstract to obtain content relevant to this topic. Finally, a total of 110 references were cited in this paper. Of these, 19 were published after 2020, 46 in 2018–2019, and 24 in 2015–2017. The main sources of the literature include Automation in Construction (41), Computer-Aided Civil and Infrastructure Engineering (5), Engineering, Construction and Architectural Management (6), Journal of Computing in Civil Engineering (3), Sensors (3), and Advanced Engineering Informatics (3). All the literature searched is from Web of Science (WOS).

## 3. CPS: Current Status of Applications and Developments

### 3.1. Concept and Characteristics of CPS

CPS is generally considered to be the next generation of engineered systems. It is a controllable and scalable system. The abilities of computing, communication, and control are deeply integrated on the basis of information acquisition [6]. CPS is closely linked to the physical world around it and its ongoing processes [17]. The term “cyber–physical systems” was first used in 2006. “Cyber–physical systems” is from “cybernetics” [18]. In other words, today’s CPS is a continuation of the technological evolution of feedback control technology [19]. The deep integration and real-time interaction of CPS is achieved through interactive feedback between the calculation processes and physical processes, thus enabling the physical entities to be detected or controlled in a safe, reliable, and efficient way [6].

Cyber–physical systems integrate computing, communication, sensing, and actuation with physical systems to enable time-sensitive functions and interaction with the environment and human [20]. CPS is the transformation and integration of existing network systems and physical systems. Through this integration, the CPS enables real-time collaboration with the physical system. The physical system collects data through the CPS, passes the data to the information processing layer according to the service requirements, and accomplishes the tasks through data processing and feedback control techniques [6]. Therefore, this complex system must possess trustworthiness, which is lacking in many of today’s cyber infrastructures [7].

It is worth noting that the CPS does not directly reference either implementation approaches or application. Instead, it focuses on the fundamental intellectual issues that combine the engineering traditions of the cyber and physical worlds [18]. This nature of CPS allows for different implementations in different domains. However, this also leads to the need to reconstruct and discuss the implementation of CPS when it is applied in a different domain. In addition, some researchers have argued that a digital twin is also a specific form of CPS that refers to a real-time digital replica of a physical process including all information that could be useful during all lifecycle stages [21]. Consistent with a CPS, a digital twin can enable feedback from a digital model to a physical entity in the real world [22]. Currently, in the manufacturing industry, digital twins can be used to effectively verify system performance at the CPS design stage through semi-physical simulation [23,24]. It is also possible to combine cyber and physical systems to achieve rapid change and rapid configuration of physical systems [25]. The digital twin semi-physical simulation can be well combined with BIM to simulate the problems existing in the construction stage and quickly verify the effectiveness of the construction scheme.

### 3.2. Common Architecture of CPS

**Figure 2:**
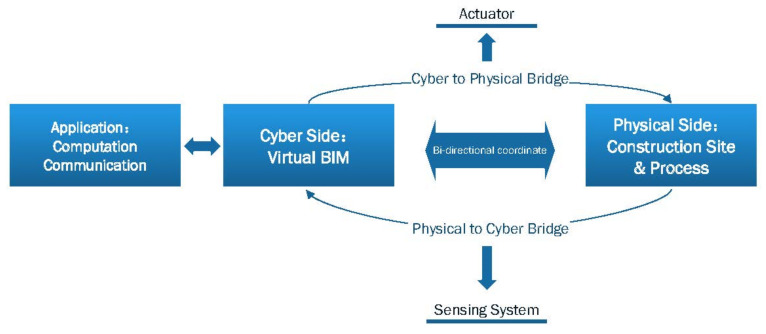
Architecture of CPS.

Figure 2 shows the common architecture of CPS in the construction industry. CPS architecture has several important components as follows: sensing system, physical world, networks, actuator, virtual model, application, and users.

First, CPS is a system that integrates on the basis of existing physical, network, and computer system architectures. In addition, abstraction and modeling of communication, computational, and physical dynamics at different time scales and sizes are required to build CPS architectures [6]. In the construction industry, in order to obtain information on the construction process and the resources involved, appropriate sensing devices need to be deployed. These sensing methods need to provide the relevant information required for CPS applications [26].

The actuator network in CPS should consist of multiple actuator units and control nodes. The control nodes are responsible for receiving commands and sending them to a specific actuator in order to adjust and control certain physical properties of the physical world [6]. In the construction industry, the selection of the actuators requires particular attention due to the lack of automation in the construction process.

The information system is the core of the CPS. For the construction industry, the virtual model of the building is the core of the information system. The integration of digital models and physical construction processes is now showing great promise to improve the productivity and safety of the construction process through resource tracking and activity monitoring [27]. Digital models, or to be more specific, building information modeling (BIM), BIM has the ability to store the full lifecycle information of a facility. It contains a digital representation of the physical facility and can be used as a platform for visualizing and monitoring the status of construction activities. The information obtained by the sensing system in the CPS is visualized in the BIM and stored in the corresponding digital model. The integration of BIM into CPS has been demonstrated in several studies [28,29,30]. It should be noted that there is a natural rationale for using BIM as the virtual end of CPS. This is determined by the characteristics above listed of the BIM itself. On the basis of the cyber side, the application layer can be further applied to the users to achieve greater benefits of CPS in practice. The CPS will operate under closed-loop control with full consideration of real-time capability, safety, and system performance [6]. As in Figure 1, the bridges between the cyber side and physical side consist of closed-loop control.

### 3.3. State-of-the-Art Researches

After clarifying the concept and architecture of CPS, the current status of CPS applications in the construction field needs to be summarized in the context of the AEC industry. This section summarizes the literature to obtain the sensing approach and feedback control methods adopted for CPS applications in construction sites. In Section 3.4, the limitations and knowledge gaps of the current approaches will be discussed. The current research of CPS in the construction industry can be divided into the following categories according to its purpose: safety management, energy performance analysis, structural health monitoring (SHM), etc. Table 1 shows the current CPS applications in the construction industry.

First, for safety management, CPS shows good prospects. Congwen Kan et al. [31] explored the applicability of cyber–physical systems to mobile cranes on construction sites and proposed a five-layer system architecture. This system provides advantages in managing mobile cranes by enabling bi-directional communication and coordination between the physical level equipment (cranes) and their digital models. This system can proactively monitor crane operations, provide rich feedback to crane operators, and avoid mobile crane failures and accidents. Abiola A. Akanmua et al. [32] described a cyber–physical posture training environment. In this environment, workers can practice the content of their work and reduce ergonomic risks. The proposed system uses wearable sensors, trackers, machine learning, and virtual reality to track the body. It also provides feedback control through an interactive user interface for the training of workers working in wooden frame construction. Cheng Zhou et al. [33] proposed a safety monitoring system for blind hoisting in underground constructions and metro. IoT technologies including wireless sensor positioning and tracking are used in this cyber–physical-system-based method to prevent accidents during the changing and dynamic hoisting process.

From the aspect of building performance management, Andrea Bonci et al. [28] proposed a cyber–physical system for the automated monitoring of buildings during regular operation. This approach allows for the management of unexpected or rare occurrences that were not explicitly designed, and provides better automation and flexibility for buildings. Mateus Vinícius Bavaresco et al. [34] reviewed the application of CPS in building energy performance with the human dimension. This paper developed an energy management method where humans interact with buildings. Alessandro Carbonari et al. [35] proposed an architecture of the CPS paradigm that can guide the operation management and long-term refurbishment processes of buildings.

For SHM and the structural test domain, Ruiyang Zhang and Brian M. Phillips [36] presented a CPS-based method to optimize the base-isolated structure. This approach provides semi-active control of the structure under seismic loading. The above study shows that CPS can play an important role in improving the performance of infrastructure under natural hazards. Capturing the physical behavior of the structure and linking it to a numerical model can create a cyber–physical framework to capture the response of the structural system. Xiao Yuan et al. [37] investigated the availability of CPS for temporary structure monitoring by determining the user requirements and the system design requirements. Theresa Fitz et al. [30] proposed a metamodel to describe CPS in structural health monitoring and mapped it into the industry foundation classes (IFC), an open standard for BIM.

At the level of an overall perspective, Raihan Maskuriy et al. [38,39] discussed construction 4.0 and figured out the relationship between BIM and CPS. CPS can optimize the use of BIM during the construction stage. In the meantime, using BIM as the core in the CPS can adapt BIM capabilities to improve construction lifecycle management. Daniel A. Linares et al. [40] reviewed the current state of the technologies that support CPS in the construction industry. Further development of novel sensors is still needed to collect different types of data from the construction process. From another aspect, robotics, automated vehicles, or equipment can be implemented as the actuators in the CPS. Conrad Boton et al. [41] carried out an analytical study of more than 2000 publications dealing with digitization in the construction industry based on keyword extraction and normalization methods. Based on keyword frequencies, a correlation was established between BIM as the backbone of digital construction and CPS. However, this study likewise found that the importance of CPS is currently difficult to promote due to a lack of awareness. CPS in the design stage was also discussed by Christos Tsigkanos et al. [42]. Their paper explained the application of CPS at the level of space design as dynamic cyber–physical spaces. This method extracts formally analyzable models from BIM and the static and dynamic properties of the design are checked against the formally defined requirements.

Similarly, analogous to BIM and city information modeling (CIM), CPS can also be applied to broader urban scenarios, providing a fundamental approach to the establishment of smart cities [43]. Deploying CPS in smart cities will significantly improve services at multiple levels such as health care, transportation services, utilities, safety, and environmental health [44].

### 3.4. Discussion and Current Limitations

State-of-the-art studies have shown that although CPS has been tested in the construction field, the current state of CPS still does not allow for its widespread application, especially for on-site construction processes. This problem is the result of a combination of factors. The AEC industry is traditionally characterized by a high degree of process diversity. Compared to manufacturing, the construction industry has a lower capacity to change as well as new technologies [47].

As above-mentioned in the literature, currently, CPS is more widely used in domains such as building energy management [29,48]. This is because the research objects in this domain have clear quantitative criteria; the data are computable and can be easily captured by sensors and incorporated into the management of CPS. However, the CPS required in this paper is dealing with decentralized, discrete construction processes that are difficult to quantify or capture directly. The construction process involves multiple elements, encompassing at least the workforce, materials, equipment, and the building itself. There is also a complex relationship between these four elements, and the entire construction process itself is dispersed at the construction site. Elements that are not directly adjacent in time and space may also be linked in the construction process. In addition, the construction site itself has a complex environment, which is constantly changing as the construction process progresses. It is further complicated by the fact that existing IoT or RFID methods are not sufficient to obtain sufficient site information and do not cover the entire construction site because the large size of the site requires a considerable number of sensors and complex data transmission solutions. In addition, for the RFID method commonly used in manufacturing CPS, the problem is that the tag has no read/write capability and cannot realize the concept of bi-directional coordination [26].

For construction sites, existing methods are not acceptable to contractors in terms of cost and difficulty. An example can be seen in the existing research [26]. The decentralized nature of the construction industry is a major barrier to the application of CPS. The decentralized arrangement of the construction process and the large size of the construction site present high demands on the sensors as well as cost-level barriers. In addition, the limited use of virtual models during the construction phase also affects the application of CPS.

For the specific environment of the AEC industry, it is necessary to propose a novel approach to sense the construction site in order to further develop the application of CPS in the construction process.

## 4. Computer Vision in Construction

### 4.1. Computer Vision

Computer vision is a technology that allows computers to learn to “watch” and to have human-like “vision” capability. Computer vision methods allow computers to extract higher-level, abstracted information from huge amounts of image or video data. Currently, there are many different tasks in the field of computer vision such as object detection, instance segmentation, semantic segmentation, object tracking, etc. depending on the input image information and the required output information. After the concept of deep learning was introduced, computer vision methods based on deep learning, especially convolutional neural networks, have been rapidly developed. Deep learning and convolutional neural networks have brought a great revolution in the field of computer vision, and it is this revolution that provides a far better foundation for the application of computer vision in construction sites. Convolutional neural network-based methods now play an important role in many fields including object detection, instance segmentation, and tracking.

After reviewing the computer vision methods, it was found that object detection and the instance segmentation method can benefit computer vision-based construction site sensing. In addition, tracking methods for various types of objects are equally important.

**Figure 3:**
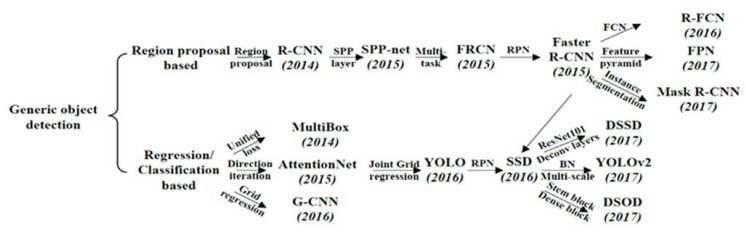
Object detection: one-stage (region proposal based)/two-stage (regression/classification based) [49].

Object detection is a method to precisely estimate the concepts and locations of objects contained in each image to gain a complete image understanding. It is able to provide valuable information for the semantic understanding of images and videos [49]. Currently, there are mainly two different approaches to object detection/instance segmentation methods as Figure 3 shows. One is a series of methods that are evolved based on the regional proposal method proposed by R-CNN [50]. This type of method is also known as the two-stage method because its detection process first generates a group of regional proposals, and then classifies each regional proposal afterward. The method that completes location detection and classification in the same process is called the one-stage method.

These two types of methods each have different advantages and disadvantages. In general, the two-stage approach performs better at the level of mAP and object location accuracy; however, it consumes more computing capability and time. Although tricks can be used to make it better in real-time, overall, the one-stage method can perform the detection task more quickly with better real-time performance [49].

The development of the two-stage method has a clear line of succession. From R-CNN to Mask-R-CNN, the development is established based on the previous generation of technology. The two-stage method includes R-CNN [50], SPP-net [51], Fast-R-CNN [52], Faster-R-CNN [53], Mask-R-CNN [54]. The one-stage method includes SSD [55] and Yolo (from v1 to the latest Yolo v4) [56,57,58,59].

Instance segmentation can be considered as a further extension of object detection. In object detection, a specific object in an image is localized by a rectangular bounding box. However, in this localization method, it is difficult to specify the exact location of the object in complex states. In practical applications, object boundaries are also important information. The algorithm represented by Mask-R-CNN [54] achieves instance segmentation by mask generation based on the above-mentioned object detection. Mask-R-CNN adds a branch for mask prediction by building on the structure of the original Faster-R-CNN. Since then, the author of Mask-R-CNN has continued to improve the algorithms at the instance segmentation level and built RetinaNet [60], TensorMask [61], PointRend [62], and other algorithms to further improve the performance of instance segmentation in various datasets.

### 4.2. Computer Vision-Based Construction Site Sensing

Currently, with the increasing demand for digitalization and intelligence in the construction industry, the research on the application of deep learning-based computer vision methods in construction projects has been developing rapidly. The construction site is complex and the current application of computer vision in construction sites generally selects a specific entity or several interrelated entities at the construction site for the corresponding site sensing and information acquisition. Researches carried out on the construction site vehicles, construction machinery, and workforce detection show that the use of computer vision methods can effectively obtain construction site human–machine information, and this information will play a key role in intelligent management for construction.

**Figure 4:**
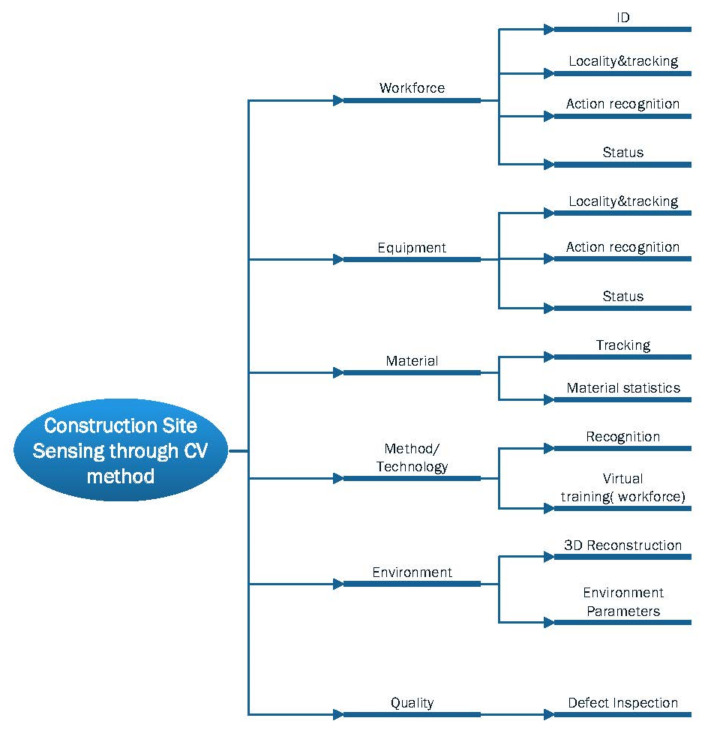
Computer vision-based construction site sensing.

Depending on the selected construction site entity, the sensing of the construction site can be divided into the following components: workforce, equipment, materials, construction methods and technology, environment, and quality. The CV algorithm can also be further categorized based on the categories of information acquired when applied to these different entities, as shown in Figure 4. The current study focuses on workforce and equipment as well as on-site construction activity because the state of human–machine at the construction site is the key to construction management. CV methods are less commonly used in materials, environment, and quality areas. The following section will categorize and organize the literature in these areas and present the current status of their research.

#### 4.2.1. Workforce

**Figure 5:**
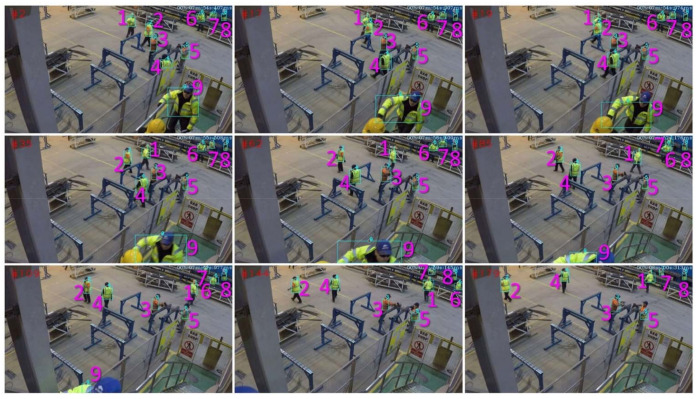
Multiple worker tracking [64].

The workforce is the most important factor on the construction site. Currently, the level of automation in the construction industry is relatively low, and the need for labor is still high. This also means that the management of the workforce is one of the most important out of all aspects of site management. The productivity of workers on site is always a concern for managers. In addition, personnel safety is another noteworthy issue. As shown in Table 2, computer vision can enable the acquisition of information about the whole process of construction at multiple levels for the on-site workforce. Figure 5 is a typical worker tracking method. The tracking algorithm can give a fixed number to the workers in the construction scene, and keep tracking the workers during the construction process. Reidentification (ReID) is a noteworthy novel algorithm for worker tracking. This algorithm combined with multiple cameras can effectively acquire the 3D coordinates of workers while continuously maintaining tracking [63]. Currently, the main barriers to worker detection on construction sites exist in the complex environment and cluttered background of construction sites. In addition, the similarity of workers in appearance (due to workwear/PPE wearing) also makes it more difficult to identify/trace workers on site [64]. 

#### 4.2.2. Equipment

**Figure 6:**
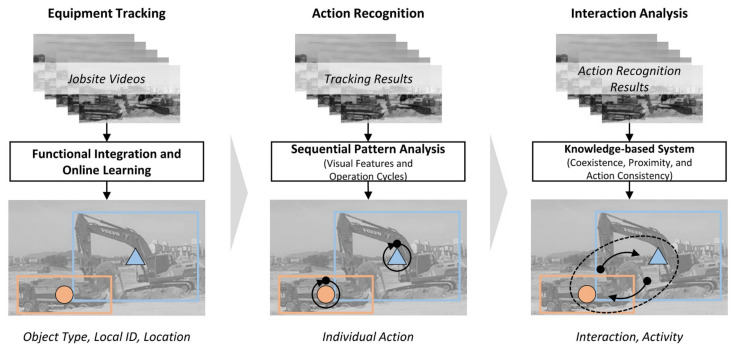
Equipment tracking, recognition and interaction analysis [88].

**Figure 7:**
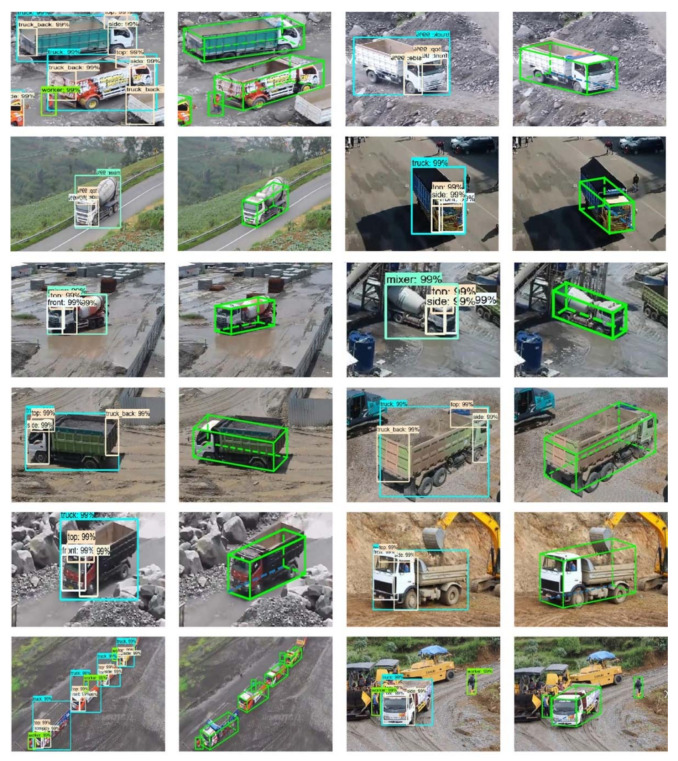
3D Heavy vehicles in 3D relationship recognition [89].

There is a wide variety of equipment at construction sites, and its tracking and monitoring play an important role in site management. For heavy equipment on-site such as tower cranes, their operational status has a major impact on both site safety and productivity. Figure 6 shows a method for obtaining the productivity information of mechanical equipment on the construction site based on computer vision and LSTM. This method can judge the relationship between associated equipment while obtaining the type and location. The method in Figure 7 pays more attention to the acquisition of spatial relationships. This method uses the known information of the vehicle to infer its three-dimensional spatial relationship based on the two-dimensional image. Table 3 shows the information acquisition of construction equipment from existing CV-based studies.

#### 4.2.3. Material

**Figure 8:**
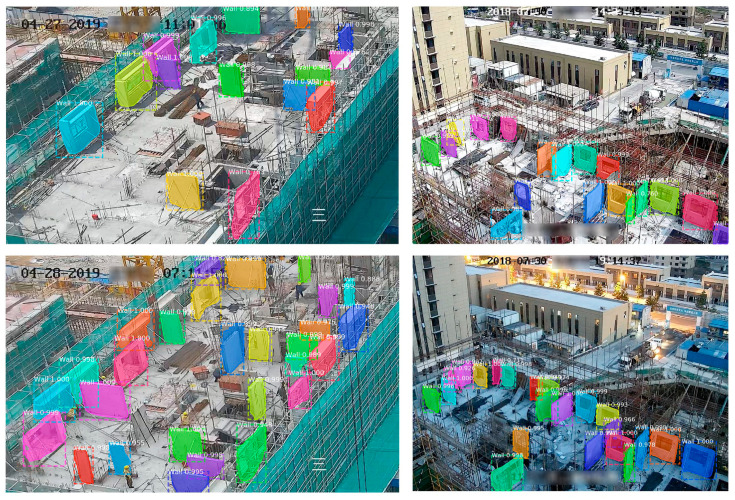
Detection and tracking of pre -cast components [90].

Identifying and tracking construction materials is essential in the construction process. Management of the transportation, lifting, and installation of building materials requires access to a variety of information about their location, quantity, and condition. The use of computer vision on construction sites can provide the information needed for management in a non-intrusive way. In busy and complex construction sites, the non-intrusive, low-cost advantages of visual methods can be better demonstrated. Table 4 provides existing methods to obtain quantity and status information for some of the building materials. However, current research in this area is still insufficient. Currently, with the implementation of a pre-cast structure, the percentage of pre-cast components in the on-site construction materials is increasing. The identification and tracking of pre-cast components can also perform well in computer vision [90]. Figure 8 shows the tracking of a precast component hoisting process by the computer vision method.

#### 4.2.4. Construction Activity/Method

Construction activity is the basic component unit of the construction process. For CPS, to achieve a digital representation of the production process, the identification of on-site construction activity is necessary. However, construction activity involves interactions between multiple entities and is more complex than the recognition of a single entity. Visual methods for complex construction activity still need further development. Table 5 shows the current research for construction activity recognition.

#### 4.2.5. 3D Reconstruction

The object detection method only acquires the position of the object in 2D image pixel coordinates, but both the implementation of construction management and the establishment of CPS need the actual 3D coordinates of the object as the basis. Therefore, the 3D reconstruction of the construction site is very necessary. There are a number of methods available to achieve this goal. Table 6 shows the current 3D reconstruction methods.

#### 4.2.6. Damage Identification

The detection of damage on structural components of existing buildings or buildings under construction based on computer vision methods has been proven to be an effective method. However, there is still no effective method for construction quality inspection during construction. This field is still underdeveloped. Table 7 shows the current research for damage identification.

#### 4.2.7. Safety Management in Construction Site

In general, although there have been studies on construction safety management based on computer vision, the focus of this research is still at the level of identifying image features (e.g., hard hat, etc.) of the construction site rather than a systematic approach to management. The combination of CPS can better accomplish this task. Table 8 shows the current research for safety management.

### 4.3. Limitations

The review above shows that computer vision is currently being tested in the construction industry. As an emerging cross-discipline, this field is developing very rapidly. However, the application of computer vision on construction sites is still in its infancy. At present, researchers are still devoted to the exploration of computer vision in the construction field. Although these methods have accomplished their objectives using computer vision methods in construction site scenarios, they lack a systematic solution and do not form an effective closed-loop control, and some of the studies have only accomplished the initial goal of identifying and acquiring information, but not the subsequent reconstruction of construction scenarios or the simulation and analysis of the construction process. Similarly, there is a lack of research on how to further utilize the information extracted from the images by computer vision methods to systematically improve the intelligence of the construction process. Among the above literature, only [112] conducted a construction safety analysis based on the relationship between worker, equipment, and materials at the construction site. The method proposed in this paper relies mainly on expert scoring, which is subjective and needs further validation for applicability between different scenarios.

This knowledge gap limits the application of computer vision in construction. There is a need for an architecture that can effectively apply the information obtained through CV to better facilitate relevant research.

## 5. Proposed Solution

### 5.1. CV-Based Construction Site Sensing and CPS

During the construction stage, CPS creates a looped connection between stakeholders that provides access to virtual models via mobile devices and facilitates decision-making [3]. CPS is the product of integration of heterogeneous systems [6]. To a large extent, the image information collected by the on-site camera system and the high-level, abstract geometric information and component information stored in the BIM model is heterogeneous data. Using computer vision methods to extract the required abstract information from the image data, transform the heterogeneous data into computable and storable homogeneous data, and apply it to the BIM model located on the cyber side is an approach that is completely consistent with the concept of CPS. Currently, there have been attempts to combine computer vision with BIM. Camera-based unmanned aerial systems (UAS) already use computer vision algorithms to collect and process inspection data, and a bridge information model (BrIM) to store and manage all relevant inspection information [114]. This can be considered as a prototype of a construction process CPS, which includes the sensing processes and information core of the CPS.

Therefore, after completing the review of the current state of application of CPS and computer vision methods on construction sites, this paper concludes that implementing a relatively low-cost construction site perception system based on computer vision methods and applying it to the construction of CPS architectures is a very promising option. Zhenhua Zhu et al. [69] found that the on-site videos contain rich information for site engineers and managers to analyze construction productivity, monitor construction progress, inspect job site safety, etc. There are several reasons for this choice: first, camera systems are inexpensive and are now widely used at construction sites. However, in most construction sites, this valuable video data are not fully utilized. Because of the labor-intensive nature of obtaining information from these videos and images on construction sites, these cameras are generally used only as an aid to human monitoring. Since the placement of camera systems on construction sites is already a sunk cost, the willingness of contractors to accept this approach could be increased if the existing equipment could be put to greater use.

Second, camera systems cover a larger area than RFID or IoT-based sensor systems and are more advantageous for use on construction sites where the layout is dispersed. Cameras with auto-tilt systems can also be better adapted to construction sites where the location of the construction process is constantly changing, focusing on key points of the construction site.

Third, computer vision-based method is non-invasive. This solution is more flexible than invasive detection such as RFID or wearable devices and also reduces the cost of equipment placement. Because there is no need to pre-arrange on the object to be detected, it has a better ability to respond to unanticipated situations.

Finally, the information acquired by the camera, although requiring more computing power to process, is richer than that of single-function sensors and RFID. The construction process is a complex one, and it is difficult to obtain a complete picture from a single sensor. However, vision-based image information contains information that can be used to identify and judge the construction process. Just as in traditional human-based site management, a construction worker can easily judge the construction process visually. The goal of the computer vision approach is precisely to give computers processing power similar to human vision.

The 3D laser is also a feasible method on construction sites. 3D point cloud is more detailed on-site information. However, the cost and detection range make it difficult to use for construction scenarios.

Table 9 shows the advantages and limitations of cameras, common sensors, and 3D laser in practical applications. It can be found that the camera may have greater advantages in certain scenarios.

In crowded and busy construction sites, vision-based methods of acquiring information have a unique advantage over other methods. With careful planning, hundreds of RFID tracking tags can be replaced by a small number of cameras placed on the construction site, significantly reducing the cost of information acquisition [115].

### 5.2. Architecture of CV-Based CPS for Construction Process Monitoring

**Figure 9:**
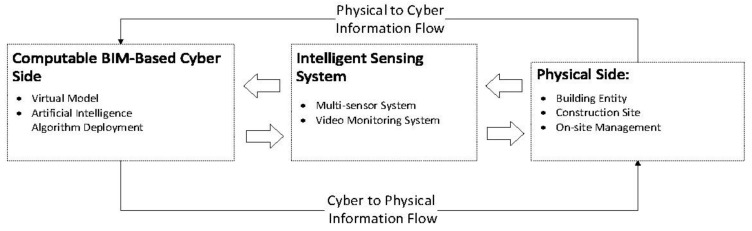
Overall framework of the construction process CPS.

**Figure 10:**
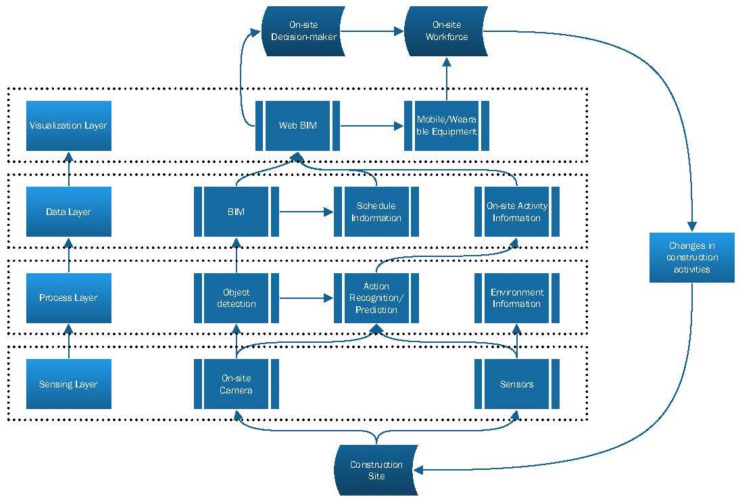
Four layers of the construction process CPS.

Based on the above reasons, the following CPS framework is proposed in this paper as a solution to the current difficulties in applying CPS in the construction field. The CPS architecture proposed in this paper places more emphasis on the mirror mapping between physical and digital entities on the construction site, and aims to establish a virtual construction site as well as a mirror relationship and synchronous mapping from a physical to virtual construction site through site sensing. First, Figure 9 is the overall framework of CPS for the construction process. The composition and operational flow of this system are demonstrated in Figure 10. The four layers of this system are as follows:

(1) Sensing layer: The function of this layer is to obtain primary information from the construction site and consists mainly of surveillance cameras installed at the construction site. At the same time, 3D lasers can be added to supplement the information acquisition channel in key areas. Other sensors can also be integrated into the system as additional information to obtain a more detailed site perception. The camera layout needs to cover as much of the main construction area as possible, with multiple viewpoints for areas where the workforce and equipment are concentrated as well as an overall view of the construction site.
(2) Process layer: Although visual data are rich in information, it is also more difficult to process than other methods. Especially for the complex environment of a construction site, the information required by the system is at many different levels, and the extraction of high-level, abstract, and computable information from the visual data requires a combination of different processing methods. Category and location detection, edge detection, and activity/state recognition of workforce and equipment require a composite approach. This layer is the computationally intensive part of the system, and the extraction of the various features in images needs to be considered in order to minimize the computational stress.
(3) Data layer: The function of the data layer is to integrate, archive, and store the original data (BIM, drawings, mechanical equipment parameters, etc.) with the real-time data from the site during the construction process. The original BIM needs to be matched with the real-time site conditions to derive the actual construction schedule information. In addition, the construction activity information obtained on-site also needs to be integrated with BIM to be used in subsequent analysis.
(4) Visualization layer: Due to the characteristics of the construction site itself, the closed-loop control of the site must be carried out by the workers on the construction site. The current building construction process is still in a human-dominated state. Construction sites also lack mechanical devices that can provide direct feedback control. Therefore, the feedback of information is mainly through two ways of the visualization layer. First, for the information directly related to construction site personnel such as safety information, this type of feedback is provided directly through the mobile devices or wearable devices of the on-site workers. The CPS environment typically includes humans, and humans function in a different way to the other components of a CPS. The architecture must support a variety of modes of human interaction with CPS to include the human as the CPS controller or partner in control [20]. The best way to achieve human–computer interaction on site is based on the visualization of mobile/wearable devices. In this layer, commands and instructions can be given to workers on-site based on visual 4D models and mobile devices, providing feedback from the virtual to the physical side.

For the information that must be involved at the decision-making level such as productivity management, schedule control, and construction quality, the feedback control is provided through visual BIM presented directly to the decision-maker. This feedback mode is mainly determined by the current construction automation level and management mode. In the future, the construction site with higher automation can further optimize it to realize more intelligent and automatic decision-making and feedback.

This four-layer structure above provides a generalized framework for the construction process CPS. Considering the actual situation of the construction site, except for a few mechanical equipment, the construction site lacks actuators. This phenomenon is caused by the level of automation on site. This framework incorporates person–system and person–environment interactions into the system through a visualization layer that provides instructions or warnings to the on-site workforce as feedback. This framework is only a preliminary idea to achieve the co-application of CV and CPS. It is expected that this framework will provide ideas for the application of computer vision on construction sites and encourage the combination of CPS and CV methods to achieve smarter construction sites.

### 5.3. Limitations and Current Barriers

Although computer vision-based site sensing has many of the above-mentioned advantages, its current application on construction sites also has certain limitations. In order to overcome these limitations, there are still obstacles that need more novel technology to overcome.

First, the layout of the construction site frequently changes with the construction progress. From the time that equipment enters, the layout of the site will be constantly adjusted as the construction phase progresses. The working surface/temporary yard/temporary facilities will also change constantly during construction, and the number of tower cranes will also increase or decrease as construction proceeds. As cameras are often placed on construction equipment/facilities, these physical changes to the construction site will lead to additional challenges to the camera system and it will be difficult to achieve complete monitoring. Adjustments would also create more workload.

Second, the issue of occlusion during construction is also a major impediment to the application of visual methods. The problem of occlusion is multi-layered, starting with the problem of objects in front blocking objects in the rear in a localized area of the work surface. This can be solved by placing the camera at multiple views. The other level is the macro-level blocking of the existing work surfacing over the completed floor. Ensuring adequate views will greatly increase the number of cameras that need to be placed. The reliability of computer vision methods for extracting information is also an important issue.

Finally, visual methods for processing on-site video require more computing power. In addition, there is no appropriate method for calculating the different layers of information that need to be extracted, which leads to repetitive computation and wasted computing power. For the large-scale application of this CV-based approach, the above-mentioned knowledge gaps must be filled. Better solutions are still waiting to be proposed.

In addition, the current construction site automation is still at a low level, and a large amount of work needs to be done manually by the on-site workforce, which leads to a great limitation of the control method from the information side to the physical side of the CPS. Currently, feedback control can only be achieved through visualization and instructions to the on-site workforce to transfer information from the cyber side to the physical side. Although this approach enhances the digital and intelligence level of the construction site, there is still a gap between this approach and the automation control expected from CPS. This is a compromise due to the complexity and decentralized nature of the construction site. The solution to this problem is to look forward to the further development of automated construction, so that actuators that can be connected to the cyber-side on the construction site can play a major role.

## 6. Conclusions

In this paper, an overview of the state-of-the-art computer vision-based construction site sensing methods is presented to further expand the application of CPS in the AEC industry. Based on the review, in-depth issues affecting the application of CPSs on construction sites and current knowledge gaps are stated. It was found that computer vision is an excellent method of construction site perception. The potential of CPS can be further stimulated by providing rich information from on-site perception using computer vision methods. The main contribution of this paper is to propose a CPS architecture using computer vision as the main information acquisition method based on the literature review. This architecture innovatively introduces computer vision as a sensing method of a construction site, and realizes low-cost and non-invasive information acquisition in complex construction scenarios. This architecture can digitize the construction process and provide an information base for intelligent construction management. This method can be used as an important supplement to on-site sensing to further promote the automation and intelligence of the construction process. Finally, the limitations and possible obstacles to the application of the above method are also discussed.

## Figures and Tables

**Table 1:** CPS in construction site.

Reference	Sensing Method	Objectives	Feedback	Environment	Publish Time
[37]	Multi-sensor (load; switch; accelerometer; displacement)	Temporary structures monitoring	Visual feedback/mobile devices	Test environment	2016
[31]	Sensor and RFID	Mobile cranes	Visual/control	No case study	2018
[28]	Sensor and equipment information	Building performance	Equipment switch	Test environment (single room)	2019
[36]	Sensors	Semiactive control of a base-isolated structure	Actuator	Structural tests	2019
[33]	(Camera; ultrasonic positioning; laser ranging; wind speed etc.)	Blind hoisting safety monitoring	Simulation and visual display	On-site case study	2019
[32]	Vision and wearable IMU	Construction workers training	VR device	Evaluation test	2020
[45]	Multi-sensors	Construction safety	Risk warning and visualization	On-site case study	2020
[46]	IMUs and UWB	Improve mobile crane safety	Visual and audio through the tablet	Site implementation	2020
[29]	Sensor network	Building performance	Visual display	Case study	2020

**Table 2:** Computer vision applications in the workforce.

Reference	Algorithm/Method	Research Object	Environment	Publish Time
[65]	HOG/Color histogram and SVM	Location of workers	On-site video	2012
[66]	HOG & Color	2D detection of workers	On-site video	2013
[67]	HOG	Localization of workers	On-site videos	2016
[68]	SVM	Identifying mason workers’ poses for safe and productive	Masonry construction test scenario	2017
[69]	HSV & SVM	Integrated workforce detection and tracking	Jobsite videos	2017
[70]	CNN & LSTM	Worker behavior detection	Test environment	2018
[71]	Motion/Geometry/Template matching	Across views tracking for worker	Test environment/offsite manufacturing facility	2018
[72]	CNN	Workforce activity assessment	Image database (workers installing reinforcement)	2018
[73]	CNN	Worker posture	Onsite image	2018
[64]	Color & SVM	Worker tracking/prediction	Experiments	2019
[74]	HOG & Entity matching	Worker tracking	Test videos	2019
[75]	Faster R-CNN	Detection of construction workers	Image from actual construction sites by movable camera	2019
[76]	Single RGB camera-based 3D motion capture	Worker fatigue status	Test environment	2019
[63]	ReID	3D localization of workers	Video from on-site multiple-camera system	2021

**Table 3:** CPS on a construction site.

Reference	Algorithm/Method	Research Object	Environment	Publish Time
[77]	Morphological methods	Tower cranes identifying	Image Data	2012
[78]	IPPHT	Tower crane hoist tracking	On-site image	2013
[79]	Gaussian background modeling algorithm	Tower crane tracking	On-site Image	2014
[80]	HOG	excavator skeleton estimation	On-site video	2017
[81]	Trigger-based approach	Over-height vehicle detection	Video data in 6 various locations (involving obstructions)	2018
[82]	Faster R-CNN	Obtaining spatiotemporal information of vehicles on bridges	Vehicles in the Bridge Traffic Environment	2018
[83]	CNN+ Double-layer LSTM	Earthmoving excavators action recognition	On-site data	2019
[84]	CNN	Excavator pose	Image data	2019
[85]	Mask R-CNN	Tower crane hoist safety	On-site environment	2019
[86]	OAFF-SSD	Vehicle detection	UAV data	2020
[87]	HG-CPN	Excavator pose	Image data	2020
[88]	CNN & LSTM	earthmoving equipment	Video dataset	2020
[89]	Range estimation in the monocular 2D vision	3D relationship recognition for heavy vehicles	Image dataset	2020

**Table 4:** Computer vision applications in materials.

Reference	Algorithm/Method	Research Object	Environment	Publish Time
[90]	Mask-R-CNN	Precast wall	On-site video	2021
[91]	Threshold segmentation and canny edge extract	Rebar (bandaged)	Only in test image	2017
[92]	SPBLM and FSM	Rebar diameter, spacing, and quantity	Test environment	2018
[93]	Binary thresholding for background segmentation	Extrusion quality monitoring for robotic construction	Test environment	2019
[94]	LBPs and SVMs	Tile area calculating	On-site experiment	2020

**Table 5:** CPS on a construction site.

Reference	Algorithm/Method	Objectives	Environment	Publish Time
[83]	CNN+ Double-layer LSTM	Earthmoving excavators action recognition	On-site data	2019
[95]	CNN, HMM, GMM, SVM	Earthmoving equipment activity analysis	On-site video	2019
[88]	CNN and LSTM	Earthmoving equipment productivity	Video dataset	2020

**Table 6:** Computer vision applications in 3D reconstruction.

Reference	Algorithm/Method	Objectives	Research Object	Environment	Publish Time
[96]	Visual feature matching	Vision-based reconstruction	Environment	Stereo images	2011
[97]	Camera calibration and triangulation	3D vision trackers	Trajectories of on-site entities	Indoor test facility	2012
[98]	Review	3D terrain reconstruction	Environment (terrain)	On-site images	2016
[99]	Combination of different computer vision algorithms	Construction state recognition	Interior construction environment	Indoor images of sequence	2018
[100]	Vision-based reconstruction	Semantic construction reconstruction	Construction site	On-site video	2018
[101]	SSD + KCF	Automatic matching between views	Excavator	On-site test	2018

**Table 7:** Computer vision applications in damage identification.

Reference	Algorithm/Method	Objectives	Research Object	Environment	Publish Time
[102]	UVA, PSVM	Automatic pavement cracking detection	Pavement cracking	UAV collected images	2016
[103]	Faster-R-CNN	Detecting multiple damage types	Steel/concrete damages	Image data	2018
[104]	FCN	Classification and detection of tunnel lining defects	Tunnel lining defects	Image data	2018
[105]	CNN + IoT	Bridge crack detection	Concrete cracking	Image data	2018
[106]	Faster R-CNN	Automatic damage detection	Masonry building damage	Image data	2019

**Table 8:** Computer vision applications in safety management.

Reference	Algorithm/Method	Purpose	Research Object	Environment	Publish Time
[107]	Image-Skeleton-Based	Identifying unsafe behaviors of workers	Worker’s behavior	Test environment	2017
[108]	Faster-R-CNN	Hard hat wearing	Workers; hard hat	Construction site images	2018
[109]	Faster-R-CNN + CNN	Determine worker’s harness wearing when performing tasks	Workers; safety harness	Construction site images	2018
[110]	VGG-16	Transfer learning for safety guardrail detection	Safety guardrail	Only in dataset	2018
[111]	SSD	Hard hat wearing	Workers; hard hat	Construction site images	2018
[112]	Faster-R-CNN (Baidu API)/AHP and grey clustering evaluation for comprehensive risk assessment	Comprehensive risk assessment and safety prewarning	Workers, equipment and materials	Case study	2020
[113]	SVM + HOG	Pro-active warning system for crossroads at construction sites	Moving vehicle	36-h video data test	2020

**Table 9:** Advantages and limitations of cameras, common sensors, and 3D laser.

	Common Sensor *	Camera System	3D Laser (LiDAR)
Range	Commonly short	Long	Limited
Cost	Variety	Low	High
Weight	Variety	Light	Heavy
Information	Single category	Rich information	Medium
Process cost	Low	High	Medium-High
Research depth	Long-term research and practice	Further research is needed	Long-term research and practice
Work condition	Almost no limitation	Limited by illumination/visibility	Limited by reflectivity/visibility

* Refers to common sensors such as force transducer, strain gauge, displacement meters, accelerometers, temperature and humidity sensors, wind speed sensors, etc.

## Data Availability

This study did not report any data.
